# Concurrent Conditions and Human Listeriosis, England, 1999–2009

**DOI:** 10.3201/eid1701.101174

**Published:** 2011-01

**Authors:** Piers Mook, Sarah J. O’Brien, Iain A. Gillespie

**Affiliations:** Author affiliations: Health Protection Agency, London, UK (P. Mook, I.A. Gillespie);; University of Manchester, Manchester, UK (S.J. O’Brien, I.A. Gillespie)

**Keywords:** Listeria monocytogenes, listeriosis, concurrent conditions, risk factors, bacteria, ICD-10, England, Wales, research, *Suggested citation for this article*: Mook P, O’Brien SJ, Gillespie IA. Concurrent conditions and human listeriosis, England and Wales, 1999–2009. Emerg Infect Dis [serial on the Internet]. 2011 Jan [*date cited*]. http://dx.doi.org/10.3201/eid1701.101174

## Abstract

The epidemiology of listeriosis in England and Wales changed during 2001–2008; more patients >60 years of age had bacteremia than in previous years. To investigate these changes, we calculated risk for listeriosis by concurrent condition for non–pregnancy-associated listeriosis cases reported to the national surveillance system in England during 1999–2009. Conditions occurring with *L. monocytogenes* infection were coded according to the International Classification of Diseases, 10th Revision, and compared with appropriate hospital episode statistics inpatient denominator data to calculate incidence rates/million consultations. Malignancies (especially of the blood), kidney disease, liver disease, diabetes, alcoholism, and age >60 years were associated with an increased risk for listeriosis. Physicians should consider a diagnosis of listeriosis when treating patients who have concurrent conditions. Providing cancer patients, who accounted for one third of cases, with food safety information might help limit additional cases.

Listeriosis is a rare but serious foodborne disease caused by the bacterium *Listeria monocytogenes*. Three groups of persons are disproportionately affected: the elderly, the immunocompromised, and pregnant women and their unborn or newborn infants. The clinical signs of disease in these persons include septicemia, meningitis, and miscarriage. Pregnant women can transmit the infection to the fetus, for whom the result can be deadly. However, these women may not have clearly overt signs or symptoms of infection. Case-fatality rates range from 20% to 50% (*1*). The susceptibility of healthy persons to symptomatic listeriosis is substantially less than that of persons with underlying conditions.

Persons with cancer, diabetes, AIDS, and liver or kidney disease are often predisposed to severe infection and death after infection with *L. monocytogenes*. This predisposition is a consequence of suppressed T-cell–mediated immunity (*2*) caused by the condition or its treatment. Similarly, pregnant women, the elderly, and those receiving immunosuppressive therapy are also at risk because of impaired or modulated immune function.

The epidemiology of listeriosis in England and Wales has changed since 2001 (*3*). Incidence has increased (2.1 cases/million population during 1990–2000 vs. 3.6 cases/million population during 2001–2009), and more cases have been found in persons >60 years of age who had bacteremia (but not meningitis). Similar patterns have been reported in other countries in Europe (*4**–**6*). The reasons for these changes are not fully understood, but they do not seem to be caused by surveillance artifacts and are not associated with sex, season, geography, ethnic or socioeconomic differences, underlying conditions, or *L. monocytogenes* subtype (*3*). We have showed that the increase occurred in persons with cancer or other conditions whose treatment included acid-suppressing medication (*7*). In view of recent trends, we examined national surveillance data for England to quantify the role of concurrent conditions in persons with listeriosis and stratified these conditions to examine risks for persons >60 years of age.

## Methods

The Health Protection Agency Centre for Infections has coordinated national surveillance of listeriosis in England and Wales since 1990. Cases are included in the system by voluntary referral of cultures to the national reference laboratory or by electronic reporting of confirmed cases from local laboratories. Clinical data, including details of patients’ concurrent conditions, are subsequently sought from the consultant clinical microbiologist involved in the care of the case-patient. Microbiologic data from local and reference laboratories and clinical and risk factor data are linked for each case, deduplicated as necessary, and stored in a bespoke Microsoft Access database (Microsoft, Redmond, WA, USA) Access database.

A case of listeriosis is defined as a person with clinically compatible illness and from whom *L. monocytogenes* was isolated from a normally sterile site. Cases are subsequently classified as either non–pregnancy-associated (persons >1 month of age) or pregnancy-associated (a maternal–fetal or maternal–neonatal pair; such pairs were considered a single case). In this study, we included non–pregnancy-associated cases reported from laboratories in England for which a clinical questionnaire was available and showed that at least 1 reported concurrent condition was present. We included cases reported during April 1, 1999–March 31, 2009 because denominator data were arranged by fiscal years. These cases included sporadic cases and cases that were identified as being part of common source foodborne outbreaks.

Authors (P.M. and I.A.G.) reviewed each reported concurrent condition and assigned an International Classification of Diseases, 10th Revision (ICD-10) (*8*), code when appropriate. Rules for assigning codes were developed at the outset to ensure standardized coding throughout the study (Technical Appendix). These rules were validated by a third author (S.J.O.), a clinically qualified investigator, who also reviewed any coding disparities. Counts were calculated of all persons and those >60 years of age for each ICD-10 chapter (ICD-10 codes are aggregated into 22 chapters) and subgroup (within each chapter).

Hospital episode statistics finished consultant episodes (FCE) data, which were aggregated by ICD-10 code, age group (0–14 years, 15–59 years, 60–74 years, and >75 years), and fiscal year, were obtained from the Health and Social Care Information Centre (*9*) and used as denominator data. These data describe episodes of continuous admitted patient care under a specific consultant for National Health Service hospital inpatients in England, and a primary diagnosis is assigned to each episode by using ICD-10 coding. To ensure reliable confidence intervals (CIs), we calculated incidence rates/million FCEs and 95% CIs for each ICD-10 chapter and subgroup in which there were >10 cases. Two ICD-10 chapters not used by hospital episodes statistics to code primary diagnoses, external causes of morbidity and mortality (V01–Y98) and codes for special purposes (U00–U99), were not considered. Relative risks (RRs) and corresponding 95% CIs were calculated as appropriate when >10 cases were reported for a concurrent condition subgroup or chapter. Analysis was then repeated for case-patients >60 years of age.

Data were stored, manipulated, and summarized by using Microsoft Access, and incidence rates and RRs were calculated by using Microsoft Excel. Differences in proportions and changes in proportions over strata were assessed by using the χ^2^ test and the χ^2^ test for trend, respectively.

## Results

A total of 1,239 ICD-10–coded concurrent conditions were reported by 1,413 case-patients with non–pregnancy-associated listeriosis in England during April 1, 1999–March 31, 2009 (Figure). Of those patients who reported >1 underlying condition, 21 (2.2%) were identified as being part of a common source outbreak. Characteristics of case-patients with and without a completed clinical questionnaire are shown in Table 1. Overall, 9.1 cases of listeriosis/million FCEs were reported over the study period (95% CI 8.6–9.6) (Table A1). Compared with all other reported conditions, higher rates of disease were reported for the following chapters (in order of highest to lowest RR): endocrine, nutritional, and metabolic diseases (RR 5.3, 95% CI 4.2–6.6); neoplasms (RR 4.9, 95% CI 4.4–5.5); mental and behavior disorders (RR 3.1, 95% CI 2.4–4.1); diseases of the circulatory system (RR 1.4, 95% CI 1.2–1.6); diseases of the digestive system (RR 1.3, 95% CI 1.1–1.5); and diseases of the musculoskeletal system and connective tissue (RR 1.3, 95% CI 1.1–1.6) (Table 2).

**Figure:**
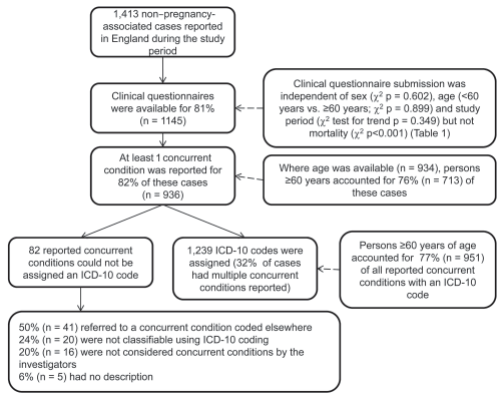
Study population and reported International Classification of Diseases, 10th Revision (ICD-10)–coded concurrent conditions for 1,413 case-patients with non–pregnancy-associated listeriosis, England, April 1, 1999–March 31, 2009.

**Table 1:** Characteristics of case-patients with non–pregnancy-associated listeriosis, England, 1999–2009*

Characteristic	No. (%) case-patients	
	CQR, n = 1,145	No CQR, n = 268
Fiscal years		
1999–2000 and 2000–2001	133 (85.3)	23 (14.7)
2001–2002 and 2002–2003	229 (89.8)	26 (10.2)
2003–2004 and 2004–2005	228 (63.9)	129 (36.1)
2005–2006 and 2006–2007	253 (81.1)	59 (18.9)
2007–2008 and 2008–2009	302 (90.7)	31 (9.3)
Sex		
M	642 (56.1)	145 (54.1)
F	503 (43.9)	122 (45.5)
Unknown	0	1 (0.4)
Age group, y		
<60	277 (24.2)	63 (23.5)
>60	866 (75.6)	193 (72)
Unknown	2 (0.2)	12 (4.5)
Status		
Died	445 (38.9)	25 (9.3)
Did not die	664 (58)	159 (59.3)
Unknown	36 (3.1)	84 (31.3)

*CQR, clinical questionnaire received.

**Table 2:** Relative risks for ICD-10 conditions for case-patients with non–pregnancy-associated listeriosis, England, 1999–2009*

Chapter and subgroup (code)	Relative risk (95% CI)	
	Versus other conditions	Age >60 y vs. <60 y
Certain infectious and parasitic diseases (A00–B99)	1.3 (0.9–2.0)	2.5 (1.1–5.9)
Neoplasms (C00-D48)	4.9 (4.4–5.5)	2.9 (2.3–3.6)
Digestive organs (C15–C26)	3.1 (2.4–3.9)	NC
Respiratory and intrathoracic organs (C30–C39)	4.8 (3.5–6.5)	NC
Breast (C50)	2.9 [2.1–4.1)	2.6 (1.4–5.2)
Female genital organs (C51–C58)	1.9 (1.07–3.5)	NC
Male genital organs (C60–C63)	2.9 (1.7–5.1)	NC
Eye, brain, and other parts of central nervous system (C69–C72)	7.3 (4.2–12.7)	NC
Thyroid and other endocrine glands (C73–C80, C97)	2.7 (2.0–3.6)	3.2 (1.6–6.4)
Lymphoid, hematopoietic, and related tissues (C81–C96)	17.6 (15.1–20.6)	2.8 (2.0–3.9)
In situ and benign neoplasms and others of uncertainty D00–D48)	0.7 (0.4–1.1)	NC
Diseases of the blood and blood-forming organs and certain disorders involving the immune mechanism (D50–D89)	1.3 (0.9–2.0)	0.8 (0.4–1.8)
Anemias (D50–D64)	1.0 (0.6–1.7)	NC
Diseases of blood and blood-forming organs (D65–D89)	2.3 (1.3–4.0)	NC
Endocrine, nutritional and metabolic diseases (E00–E90)	5.3 (4.2–6.6)	6.3 (3.5–11.2)
Diabetes mellitus (E10–E14)	11.4 (9.0–14.5)	4.9 (2.7–8.8)
Mental and behavior disorders (F00–F99)	3.1 (2.4–4.1)	1.7 (1.01–2.8)
Due to psychoactive substance (F10–F19)	12.3 (9.4–16.1)	4.7 (2.7–8.1)
Diseases of the nervous system (G00–G99)	0.6 (0.4–1.0)	NC
Diseases of the eye and adnexa (H00–H59)	NC	NC
Diseases of the ear and mastoid process (H60–H95)	NC	NC
Diseases of the circulatory system (I00–I99)	1.4 (1.2–1.6)	NC
Hypertensive diseases (I10–I15)	8.0 (5.2–12.2)	NC
Ischemic heart diseases (I20–I25)	0.8 (0.5–1.1)	NC
Other forms of heart disease (I30–I52)	2.4 (1.9–3.1)	NC
Cerebrovascular diseases (I60–I69)	0.7 (0.4–1.2)	NC
Diseases of arteries, arterioles, and capillaries (I70–I79)	2.1 (1.2–3.5)	NC
Diseases of the respiratory system (J00–J99)	0.9 (0.7–1.1)	NC
Chronic lower respiratory diseases (J40–J47)	1.8 (1.3–2.5)	NC
Other diseases of respiratory system (J80–J99)	1.7 (0.95–3.1)	NC
Diseases of the digestive system (K00–K93)	1.3 (1.1–1.5)	1.9 (1.4–2.6)
Noninfective enteritis and colitis (K50–K52)	4.3 (3.3–5.6)	2.3 (1.4–3.8)
Other diseases of intestines (K55–K63)	0.5 (0.3–0.9)	NC
Diseases of liver (K70–K77)	22.4 (17.7–28.4)	2.2 (1.4–3.6)
Diseases of the skin and subcutaneous tissue (L00–L99)	NC	NC
Diseases of the musculoskeletal system and connective tissue (M00–M99)	1.3 (1.1–1.6)	4.5 (2.7–7.3)
Arthropathies (M00–M25)	1.7 (1.3–2.2)	NC
Systemic connective tissue disorders (M30–M36)	18.3 (12.6–26.6)	NC
Diseases of the genitourinary system (N00–N99)	1.2 (0.99–1.5)	5.3 (3.2–8.6)
Renal failure (N17–N19)	12.2 (9.8–15.1)	1.7 (1.02–2.7)
Pregnancy, childbirth, and puerperium (O00–O99)	NC	NC
Certain conditions originating in the perinatal period (P00–P96)	NC	NC
Congenital malformations, deformations, and chromosomal abnormalities (Q00–Q99)	NC	NC
Symptoms, signs, and abnormal clinical and laboratory findings, not elsewhere classified (R00–R99)	NC	NC
Injury, poisoning, and certain other consequences of external causes (S00–T98)	NC	NC
External causes of morbidity and mortality (V01–Y98)	–	–
Factors influencing health status and contact with health services (Z00–Z99)	NC	NC
Codes for special purposes (U00–U99)	–	–
Total	NC	4.6 (4.1–5.3)

*ICD-10, International Classification of Diseases, 10th Revision; CI, confidence interval; NC, not calculated (for conditions with <10 cases); –, data not available.

Within these chapters, only certain subgroups showed increased rates: diabetes mellitus; malignant neoplasms of the lymphoid, hematopoietic, and related tissues; eye, brain, and other parts of the central nervous system (CNS); respiratory and intrathoracic organs; digestive organs; breast; male and female genital organs; thyroid and other endocrine glands; mental and behavior disorders caused by psychoactive substances (alcohol-related in 96% of reports); hypertensive diseases, other forms of heart disease, and diseases of arteries, arterioles, and capillaries; diseases of the liver and noninfective enteritis and colitis; and systemic connective tissue disorders (Table 2). In addition, several subgroups were associated with increased risk even when the corresponding chapter was not: renal failure, diseases of blood and blood-forming organs, and chronic lower respiratory diseases (Table 2).

Concurrent conditions were disproportionately reported for persons >60 years of age (χ^2^ p<0.001), and the rate of listeriosis for this age group (16.8/million; 95% CI 15.8–17.9) was significantly higher than that for younger persons (RR 4.6, 95% CI 4.1–5.3) (Table 2). When the RR for each chapter for persons >60 years of age (using persons <60 years of age as the reference population) was calculated, the following were associated with increased risk: endocrine, nutritional and metabolic diseases; genitourinary system diseases; diseases of the musculoskeletal system and connective tissue; neoplasms; certain infectious and parasitic diseases; diseases of the digestive system; and mental and behavior disorders (Table 2). In instances where the risk for each subgroup in persons >60 years of age could be calculated and compared with that for persons <60 years of age, all subgroups of previously identified chapters were associated with increased risk.

## Discussion

We analyzed surveillance data that included detailed denominator data by using an internationally recognized diagnostic classification system and found that a wide variety of conditions seem to increase the risk for serious infection with *L. monocytogenes*. Malignancies accounted for more than one third of conditions, and cancer patients had a 5-fold increased risk for development of listeriosis. Cancers of the blood seemed to have the greatest effect. Other high-risk conditions included diabetes mellitus; alcoholism; certain diseases of the circulatory system and the musculoskeletal system and connective tissue; noninfective enteritis and colitis; and diseases of the liver and kidney. For most high-risk conditions, the risk for infection was higher among older patients.

Case identified by the national surveillance program in England are laboratory confirmed, and most cases result in serious illness requiring hospitalization or death. Given this finding, a hospitalized population better represents the population at risk than a community population, which was used in previous studies (*10**,**11*).

The response rate to the clinical questionnaire that captured information on concurrent conditions was high and not influenced by age or sex of the case-patient, which minimized differential ascertainment of clinical data. However, we could not assess concurrent conditions for which completed clinical questionnaires were not returned. This issue indicates that the role of some conditions might be underestimated if clinicians were unwilling to return questionnaires and disclose information for certain case-patients (e.g., those with AIDS). Similarly, but less likely, reporting bias might exist if the propensity to report certain concurrent conditions were affected by the presence or absence of others conditions, or if only concurrent conditions considered relevant to *L. monocytogenes* infection were reported. Concurrent conditions were reported by the clinical microbiologist rather than by the consultants responsible for the care of the patients with concurrent conditions. These consultants might be better informed of existing concurrent conditions. However, hospital microbiologists need to be aware of such conditions to provide treatment accordingly, and questioning several consultants for each case-patient may have a negative effect on questionnaire response because questionnaires might be lost if passed between multiple consultants.

Misclassification was minimized by grouping conditions only to 3-character ICD-10 code levels. Although we acknowledge that such grouping might mask high-risk conditions apparent at the 4-character ICD-10 code level, routine surveillance data were not specific enough to further discriminate among conditions. In some instances, in which treatments were reported in the absence of relevant conditions (e.g., chemotherapy, dialysis, splenectomy), we made assumptions about the conditions requiring such treatment and coded accordingly (Table A1). Although these assumptions could inflate the incidence rates for certain conditions, they occurred relatively infrequently and were not used for treatments that could be prescribed for a range of conditions (e.g., broad-spectrum antimicrobial drugs).

Because only single-variable analysis could be performed, we could not assess the extent to which concurrent conditions were correlated, which led to the potential for uncontrolled confounding. Such method limitations might explain the high incidence associated with both diabetes and kidney disease and reinforce the need to consider these findings as highly refined hypotheses to be tested by other methods (*12*).

To our knowledge, few studies have attempted to quantify the risk for listeriosis by patient concurrent conditions. As part of a risk assessment of *L. monocytogenes* in ready-to-eat foods, researchers from the World Health Organization (WHO) and the Food and Agriculture Organization (FAO) calculated the relative susceptibility to listeriosis for certain conditions (*10*). Furthermore, risk levels for listeriosis by predisposing condition in Denmark have also been estimated (*11*). Despite differences in methods between those studies and our study, several high-risk conditions were also identified in those studies: malignancies (most notably those of the blood), kidney disease (recorded as dialysis [*10*] and renal transplant [*11*]), diabetes, alcoholism, and increased age in all 3 studies; liver disease and pulmonary cancer in the WHO/FAO study and our study; and systemic lupus erythematosus in the study in Denmark and our study (as systemic connective tissue disorders). Such commonality would seemingly validate our estimates.

The absence of AIDS as a high-risk condition in our study and its presence in both previous studies (*10**,**11*), might reflect improved treatment for HIV infection that prevents AIDS and, consequently, *L. monocytogenes* infection (*13*) or highlight a reporting bias by the consultant microbiologist. A general transplantation status, identified as a condition leading to the highest relative susceptibility in the WHO/FAO study, was not coded in our study because it is a treatment. Noninfective enteritis and colitis and certain diseases of the circulatory system were identified as additional high-risk conditions in our study but not in the previous studies. These additional conditions might be the result of improved accuracy, use of ICD-10 coding and a hospitalized reference population instead of the general population, different susceptibility calculations, or changes in the prevalence of certain conditions in the interim period (the previous studies used data from 1992 [*10*] and 1989–1990 [*11*]). However, we acknowledge that links between these conditions and listeriosis have been reported (*14**–**18*).

With these caveats in mind, our findings have implications for clinical practice and food safety policy makers. The number and diversity of conditions that appear to increase the risk for listeriosis imply that physicians working in all specialties should consider listeriosis when treating patients with concurrent conditions and provide appropriate food safety advice. Similarly, current UK government food safety advice on avoidance of listeriosis, which is delivered passively and is specific mainly for pregnant women (*19**,**20*), should be communicated actively to all high-risk groups. In prioritizing advice, policy makers should consider not only the associated risk but also the prevalence of the concurrent condition. Cancer patients accounted for more than one third of listeriosis cases, and high risks were observed for most cancer subgroups. Because we are not aware of any appropriate food safety advice that is tailored specifically for cancer patients in the UK, emphasis on this group might help to prevent further cases.

## Supplementary Material

Technical Appendix Coding Rules for Concurrent Conditions in Human Listeriosis, England and Wales, 1999?2009*.

## Appendix

**Table A1:** ICD-10 diagnosis codes for concurrent conditions among patients with non–pregnancy-associated listeriosis, showing rate of identification/FCE for each condition and comparison of all patients with patients >60 years of age, England, 1999–2009*

Chapter and subgroup (code)	All patients				Patient age >60 y		
	No. cases	FCE	No. cases/million FCE (95% CI)		No. cases	FCE	No. cases/million FCE (95% CI)
Certain infectious and parasitic diseases (A00–B99)	21	175,8220	11.9 (7.39–18.3)		10	467,930	21.4 (10.2–39.3)
Neoplasms (C00–D48)	456	1,449,3620	31.5 (28.6–34.5)		368	860,9476	42.7 (38.5–47.3)
Digestive organs (C15–C26)	72	2,665,050	27.0 (21.1–34.0)		64	1,967,238	32.5 (25.1–41.5)
Respiratory and intrathoracic organs (C30–C39)	41	965,504	42.5 (30.5–57.6)		39	727,468	53.6 (38.1–73.3)
Breast (C50)	36	1,382,014	26.0 (18.2–36.1)		22	515,774	42.7 (26.7–64.6)
Female genital organs (C51–C58)	10	587,403	17.0 (8.2–31.3)		7	345,555	NC
Male genital organs (C60–C63)	13	490,644	26.5 (14.1–45.3)		13	389,305	33.4 (17.8–57.1)
Eye, brain, and other parts of central nervous system (C69–C72)	13	196,199	66.3 (35.3–113.3)		11	58,414	188.3 (94–336.9)
Thyroid and other endocrine glands (C73–C80, C97)	47	1,957,683	24 (17.6–31.9)		37	1,053,496	35.1 (24.7–48.4)
Lymphoid, hematopoietic, and related tissue (C81–C96)	187	1,359,740	137.5 (118.5–158.7)		145	753,816	192.4 (162.3–226.3)
In situ and benign neoplasms and others of uncertainty (D00–D48)	18	2,880,269	6.3 (3.7–9.9)		16	1,286,473	12.4 (7.11–20.2)
Diseases of the blood and blood-forming organs and certain disorders involving the immune mechanism (D50–D89)	25	2,062,183	12.1 (7.85–17.9)		12	1,079,281	11.1 (5.75–19.4)
Anemias (D50–D64)	13	1,478,008	8.8 (4.68–15)		7	871,585	NC
Diseases of blood and blood-forming organs (D65–D89)	12	584,175	20.5 (10.6–35.9)		5	207,696	NC
Endocrine, nutritional and metabolic diseases (E00–E90)	83	1,831,366	45.3 (36.1–56.2)		69	804,961	85.7 (66.7–108.5)
Diabetes mellitus (E10–E14)	71	721,573	98.4 (76.8–124.1)		57	326,473	174.6 (132.2–226.2)
Mental and behavior disorders (F00–F99)	60	2,176,420	27.6 (21.0–35.5)		25	646,228	38.7 (25.0–57.1)
Due to psychoactive substance (F10–F19)	54	502,363	107.5 (80.8–140.3)		20	56,062	356.7 (217.9–550.9)
Diseases of the nervous system (G00–G99)	16	2,732,564	5.86 (3.35–9.51)		15	1,141,845	13.1 (7.35–21.7)
Diseases of the eye and adnexa (H00–H59)	1	4,392,414	NC		0	3,449,215	NC
Diseases of the ear and mastoid process (H60–H95)	0	858,208	NC		0	125,850	NC
Diseases of the circulatory system (I00–I99)	143	11,770,385	12.1 (10.2–14.3)		135	8,514,080	15.9 (13.3–18.8)
Hypertensive diseases (I10–I15)	22	306,847	71.7 (44.9–108.5)		18	193,565	93 (55.1–147)
Ischemic heart diseases (I20–I25)	29	4,079,434	7.11 (4.76–10.2)		27	2,992,521	9.02 (5.95–13.1)
Other forms of heart disease (I30–I52)	62	2,928,710	21.2 (16.2–27.1)		61	2,366,977	25.8 (19.7–33.1)
Cerebrovascular diseases (I60–I69)	10	1,656,546	6.04 (2.89–11.1)		10	1,410,024	7.09 (3.4–13)
Diseases of arteries, arterioles, and capillaries (I70–I79)	14	747,731	18.7 (10.2–31.4)		13	590,657	22.0 (11.7–37.6)
Diseases of the respiratory system (J00–J99)	66	8,441,191	7.82 (6.1–10.0)		61	4,237,898	14.4 (11.0–18.5)
Chronic lower respiratory diseases (J40–J47)	41	2,510,375	16.3 (11.7–22.2)		40	1,627,030	24.6 (17.6–33.5)
Other diseases of the respiratory system (J80–J99)	11	702,393	15.7 (7.82–28)		10	458,712	21.8 (10.5–40.1)
Diseases of the digestive system (K00–K93)	163	14607104	11.2 (9.5–13.0)		96	6288251	15.3 (12.4–18.6)
Noninfective enteritis and colitis (K50–K52)	60	1,582,958	37.9 (28.9–48.8)		33	547,430	60.3 (41.5–84.7)
Other diseases of intestines (K55–K63)	14	2,985,107	4.69 (2.56–7.87)		13	1,623,725	8.01 (4.26–13.7)
Diseases of liver (K70–K77)	72	373,347	192.9 (150.9–242.9)		38	124,000	306.5 (216.9–420.6)
Diseases of the skin and subcutaneous tissue (L00–L99)	6	2,913,757	NC		6	1,066,962	NC
Diseases of the musculoskeletal system and connective tissue (M00–M99)	97	8,106,829	12.0 (9.7–14.6)		77	3,758,159	20.5 (16.2–25.6)
Arthropathies (M00–M25)	60	3,925,790	15.3 (11.7–19.7)		52	2,016,907	25.8 (19.3–33.8)
Systemic connective tissue disorders (M30–M36)	28	171,687	163.1 (108.4–235.7)		19	67,700	280.6 (169.0–438.2)
Diseases of the genitourinary system (N00–N99)	94	8,529,328	11.0 (8.9–13.5)		73	3,392,932	21.5 (16.9–27.1)
Renal failure (N17–N19)	90	869,322	103.5 (83.3–127.3)		69	577,799	119.4 (92.9–151.1)
Pregnancy, childbirth, and puerperium (O00–O99)	0	1,2614,774	NC		0	45,099	NC
Certain conditions originating in the perinatal period (P00–P96)	0	1,928,377	NC		0	433	NC
Congenital malformations, deformations, and chromosomal abnormalities (Q00–Q99)	1	1,069,074	NC		1	38,859	NC
Symptoms, signs, and abnormal clinical and laboratory findings not elsewhere classified (R00–R99)	2	16,380,896	NC		2	7,580,878	NC
Injury, poisoning, and certain other consequences of external causes (S00–T98)	4	8,723,795	NC		1	3,213,017	NC
External causes of morbidity and mortality (V01–Y98)	0	–	–		0	–	–
Factors influencing health status and contact with health services (Z00–Z99)	1	10,621,404	NC		1	2,070,369	NC
Codes for special purposes (U00–U99)	0	–	–		0	–	–
Total	1,239	13,601,1909	9.1 (8.6–9.6)		951	56,531,723	16.8 (15.8–17.9)

*ICD, International Classification of Diseases, 10th Revision; FCE, finished consultant episodes; CI, confidence interval; NC, not calculated (for conditions with <10 cases); –, data not available.
