# Supplementary material for: Concurrent Conditions and Human Listeriosis, England, 1999–2009
Source: Emerg Infect Dis. 2011 Jan;17(1):38–43. doi: 10.3201/eid1701.101174 (PMC3204649; doi:10.3201/eid1701.101174)
Supplement: Technical Appendix — Coding Rules for Concurrent Conditions in Human Listeriosis, England and Wales, 1999?2009*. [file 10-1174-Techapp.pdf]

DOI: 10.3201/eid1701.101174

Concurrent Conditions and Human Listeriosis, England and Wales, 1999–2009

Technical Appendix

Coding Rules for Concurrent Conditions in Human Listeriosis, England and Wales, 1999–2009*

General Points

International Classification of Diseases, 10th Revision (ICD-10) codes assigned to the 3 character level (but analyses were limited to the subgroup level only).

Age has not been considered for coding unless stated in the open text underlying condition field.

Conditions were only presumed to be congenital if stated as such (see above point).

Conditions were presumed to be chronic if they could be either chronic or acute but were not described as either.

Symptoms of a condition were not coded (encephalitis, septicemia, cough).

Alcohol-related Pathologic Changes

Underlying conditions such as alcoholic, alcoholism, alcohol problem were assigned as mental or behavior diseases because of use of alcohol (ICD-10 code F10).

Conditions coded as alcoholic liver disease (K70) were, in addition, coded as mental or behavior diseases because of use of alcohol (F10).

In the absence of alcoholic, liver cirrhosis or failure was coded as fibrosis and cirrhosis of liver (K74) or other diseases of the liver (K76).

Malignancies

Cancer of the bowel was coded as malignant neoplasm of colon (C18).

Underlying conditions described as metastases, malignant tumor, cancer, cancerous growth have been coded as malignant neoplasm without specification of site (C80).

Only the primary cancer site was coded unless there was mention of a secondary site and no mention of the primary site.

Neutropenia, without any description of cause, was kept as a condition and coded as agranulocytosis (D70).

If a case with a defined malignancy was also described as being neutropenic, agranulocytosis was not coded.

Chemotherapy without any description of a malignancy or other condition was coded as malignant neoplasm without specification of site (C80).

The assumption was made that bone marrow transplant would most likely be a treatment for malignancies of the blood and, thus, in the absence of any other described conditions, was coded as other and unspecified malignant neoplasms of lymphoid, hematopoietic and related tissue (C96).

Other Chronic Diseases

Ischemic heart disease was coded as chronic ischemic heart disease (I25) unless stated otherwise. Following on from the general presumption that conditions are chronic if not stated otherwise.

Nonspecified heart disease was coded as complications and ill-defined descriptions of heart disease (I51).

Any treatments or indications of heart disease (valve replacement, fibrillations) were coded as complications and ill-defined descriptions of heart disease (I51). However, they were only coded if there was no other mention (and coding) of heart disease.

Unspecified sinusitis was presumed to be chronic sinusitis (J32).

Hepatitis B and C were coded as chronic viral hepatitis (B18) if they were not defined as acute or chronic. Unspecified and autoimmune hepatitis was coded as other inflammatory liver diseases (K75).

Renal impairment was coded as chronic renal failure (N18).

Pyelonephritis was coded as acute tubulointerstitial nephritis (N10).

Miscellaneous

Dialysis was coded as chronic renal failure (N18) because it is a condition for which dialysis is a treatment.

Although there is a causal link between renal failure and diabetes, they were coded if they were described.

Tuberculosis was assumed to be bacteriologically and microbiologically confirmed (A15).

Nonspecified anemia was coded as other anemia (D64), aplastic anemia and acquired pure erythrocyte anemia were coded as acquired pure erythrocyte aplasia (erythroblastopenia) (D60), iron deficiency anemia was coded as iron deficiency anemia (D50), and auto immune hemolytic anemia was coded as acquired hemolytic anemia (D59).

Unspecified osteoporosis was coded as osteoporosis, without fracture (M81).

Cerebrovascular accident was coded as stroke, not specified as hemorrhage or infarction (I64).

Hypertension was coded as essential (primary) hypertension (I10).

Splenectomy was coded as diseases of spleen (D73).

If splenectomy and lymphoma were described, diseases of spleen (D73) were not coded.

Lupus was coded as systemic lupus erythematosus (M32).

Chest infection (lower respiratory tract infection and bronchitis) was coded as unspecified chronic bronchitis (J42) if qualified as being chronic or bronchitis, not specified as acute or chronic (J40) if it was not further qualified.

Ascites and jaundice were coded as other diseases of the liver (K76).
